# Validation of the imperial college surgical assessment device for spinal anesthesia

**DOI:** 10.1186/s12871-017-0422-3

**Published:** 2017-09-29

**Authors:** Marcia A. Corvetto, Carlos Fuentes, Andrea Araneda, Pablo Achurra, Pablo Miranda, Paola Viviani, Fernando R. Altermatt

**Affiliations:** 1 Anesthesiology Department, School of Medicine, Pontificia Universidad Catolica de Chile, Marcoleta 367, 8330024 Santiago, Chile; 2 Surgery Department, School of Medicine, Pontificia Universidad Catolica de Chile, Santiago, Chile; 3 Public Health Department, School of Medicine, Pontificia Universidad Catolica de Chile, Santiago, Chile

**Keywords:** Medical education, Simulation-based education, Spinal anesthesia, Motion capture

## Abstract

**Background:**

Traditionally, technical proficiency for spinal anesthesia has been assessed using observational scales such as global rating scales or task specific checklists. However more objective metrics are required in order to improve novice’s training programs. The aim of this study is to validate the hand motion analysis of the Imperial College Surgical Assessment Device (ICSAD) in a simulated model of spinal anesthesia.

**Methods:**

Three groups of physicians with different levels of experience were video recorded performing a spinal anesthesia in a simulated lumbar puncture torso. Participants’ technical performance was assessed with ICSAD, a Global Rating Scale (GRS) and a specific Checklist. Differences between the 3 groups were determined by Kruskal-Wallis test with post hoc Dunn’s correction for multiple comparisons. Spearman correlation coefficient between ICSAD variables and the scores of the observational scales were calculated to establish concurrent validity.

**Results:**

Thirty subjects participated in the study: ten novice (first year residents), 10 intermediate (third year residents) and 10 experts (attending anesthesiologists). GRS scores were significantly higher in experts, than intermediates and novices. Regarding total path length, number of movements and procedural time measured with ICSAD, all groups had significant differences between them (*p* = 0.026, *p* = 0.045 and *p* = 0.005 respectively). Spearman correlation coefficient was −0,46 (*p* = 0.012) between total path length measured with ICSAD and GRS scores.

**Conclusions:**

This is the first validation study of ICSAD as an assessment tool for spinal anesthesia in a simulated model. Using ICSAD can discriminate proficiency between expert and novices and correlates with previously validated GRS. Its use in the assessment of spinal anesthesia proficiency provides complementary data to existing tools. Our results could be used to design future training programs with reliable goals to accomplish.

**Electronic supplementary material:**

The online version of this article (10.1186/s12871-017-0422-3) contains supplementary material, which is available to authorized users.

## Background

Spinal anesthesia is a critical procedure that needs to be learned promptly in the training process of residents of Anesthesiology. It represents a real challenge to traditional methods of medical education [1–3].

In this context, most of the technical proficiency in anesthesia is assessed using task-specific checklists or global rating scales applied during procedures performed in real patients. Some of these specific technical domains of performance remain underrepresented and the rates of failure and complications during training process are still an issue [4–6]. As a mean to overcome this, metrics provided by the hand motion analysis of The Imperial College Surgical Assessment Device (ICSAD have been used as complementary tools proven to objectively discriminate operator’s technical expertise in some anesthesia procedures such as labor epidural placement and ultrasound-guided peripheral nerve blockade [7, 8]. Likewise, other tracking motion devices have been used to assess operator’s performance during central venous catheter placement and endotracheal intubation [9, 10]. At the present time, no tracking motion analysis has been validated to assess operators performing spinal blocks or documenting the acquisition of technical skills.

The aim of this study was to determine the construct validity of the ICSAD as an assessment tool in spinal anesthesia by determining whether the ICSAD could discriminate operators’ proficiency. Secondly, we want to determine the concurrent validity of the ICSAD by correlating it with the scores of previously validated assessment tools [7, 11].

## Methods

The Ethical Committee of Pontificia Universidad Catolica de Chile approved the study (Protocol number 16–112/2016). Written informed consent was obtained from all participants. Subjects were categorized in three groups according to their level of experience: 10 novice (anesthesia first year residents, without previous experience in spinal anesthesia or simulation training programs), 10 intermediate (third year residents of anesthesiology) and 10 attending anesthesiologists with obstetric or regional anesthesia fellowship and at least 5 years of experience in regional anesthesia.

In order to standardize their level of knowledge, all participants reviewed a video on the technique of spinal anesthesia. Instructions and information of the protocol were prepared in a virtual platform. All participants attended the skill assessment session and performed a spinal anesthesia in a simulated torso (Gaumard® Lumbar puncture torso S411, Miami, USA) using a spinal set containing a 25-gauge spinal needle and a 21-gauge introductory needle. For purposes of analysis, we divided the procedure into (I) Preparation phase, defined as the time for preparing materials until to be ready to perform the puncture; and (II) Needling phase, defined as the time between insertion of the needle until the injection of local anesthetic and withdrawal of the needle.

**Fig. 1:**
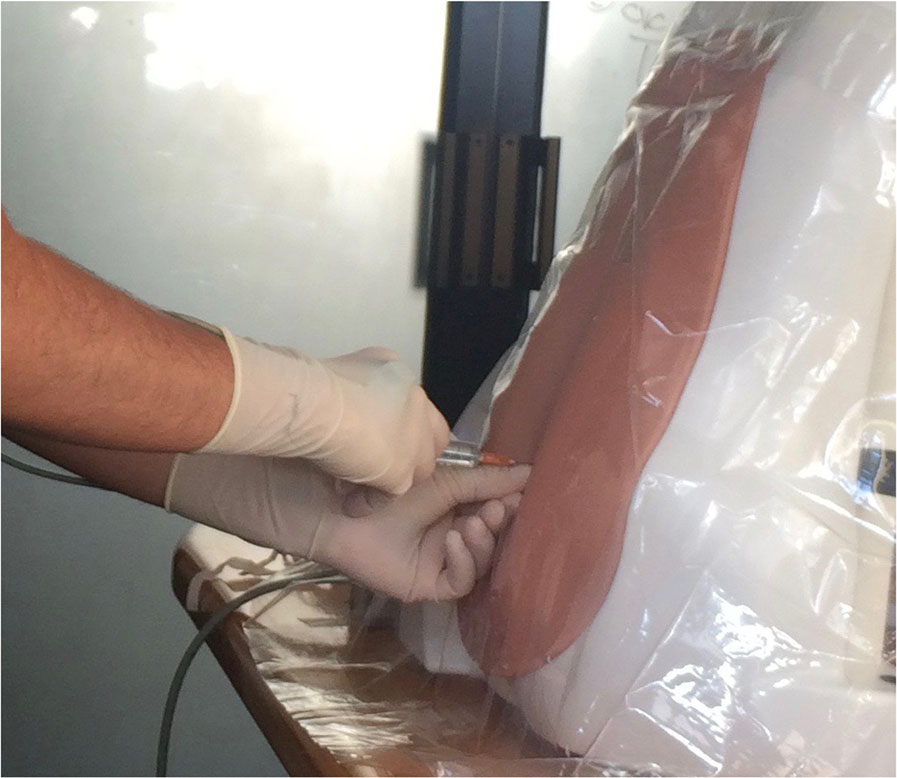
ICSAD sensors attached on the back of the participant’s hands

The ICSAD sensors were attached firmly on the back of the operator’s hands, under the gloves (Fig. 1). This device is a combination of a commercially available electromagnetic tracking system (Patriot; Polhemus, Colchester, VT, USA) and a motion tracking software program ROVIMAS developed by the Department of Surgical Oncology and Technology of The Imperial College of London [12]. The 3-dimensional Cartesian coordinates of each sensor are recorded in real time at a resolution of 1 mm and a frequency of 20 Hz. ICSAD dexterity measurements used to evaluate motion efficiency were: time taken to perform the procedure, distance traveled (total path length (TPL)) and the number of movements made by both hands.

All procedures were videotaped. In order to assure blindness, only the hands of the participants were displayed. All the participants wore gloves. Two blinded and independent evaluators reviewed all videos and rated all performances using a previously validated task-specific Global Rating Scale (GRS) (Additional file 1) [7] and a Checklist (Additional file 2) [11]. Both evaluators were anesthesiologists with experience in simulation and were trained by the primary investigator to evaluate participants using both tools. Training of the evaluators consisted of rating pre-recorded videotaped performances of simulated spinal procedures. Additionally, raters determined the number of attempts done by each participant, defined as the number of repetitions done before the needle was successfully placed at a given lumbar spine level. Every new skin puncture was considered another attempt. However, redirecting the needle without a new skin puncture was not considered an additional attempt [13].

Construct validity is the extent to which performance differs between operators possessing different levels of the construct. It is often established by assessing the performance of an expert cohort of subjects compared to a novice cohort of subjects. Concurrent validity is established comparing subjects’ performance on a new assessment with their performance on a previously validated gold standard assessment. The degree of correlation between the subjects’ performances establishes the degree of concurrent validity of the new test [14].

### Statistical analysis

Data was analyzed with the Statistical Package for the Social Sciences version 15.0 (SPSS, Chicago, IL, USA). Inter-rater reliability between GRS scores was calculated using the Cohen kappa coefficient. Given the small number of observations and consequently the limitations to apply the Central Limit Theorem, we assumed a non- normal distribution of variables. Results were presented as median and interquartile range (Q1–Q3). To explore overall differences between the 3 groups, a nonparametric 1-way analysis of variance (Kruskal-Wallis test) was made; subsequently, Dunn’s correction test for multiple comparisons was performed in order to determine the different pair. Spearman correlation coefficients between the ICSAD measurements and the validated global rating scale were calculated to establish concurrent validity of ICSAD. Following the guidelines of Cohen, a correlation coefficient of 0.1 is considered a weak or small association; a correlation coefficient of 0.3 is considered a moderate correlation; and a correlation coefficient of 0.5 or higher is deemed a strong or large correlation [15]. *P* values <0.05 were considered statistically significant.

Since a sample size calculation was not done a priori, we decided to perform a Post Hoc power analysis using a rank transformation of TPL (rank-TPL). As Rank-TPL distributes normal according to Shapiro Wilk test, it eases the analysis of variables that comes from skewed distributions and allows to determine the effect size in standard deviations (SD). Therefore, considering an effect size of 1.5 SD for rank-TPL, having a sample size of 10 subjects for each category and a significance level of 0.05 (two tailed) we obtained a power of 86% to detect a difference. These findings are consistent when we analyzed the groups for rank-TPL with One-Way ANOVA parametric test and Bonferroni correction.

## Results

A total of 30 subjects were assessed. Demographics and number of spinal anesthetic procedures previously performed are described in Table 1.

**Table 1:** Demographics

	Novices (*n* = 10)	Intermediates (*n* = 10)	Experts (*n* = 10)	*P* value
Age (y)	25.4 (23–31)	30.3 (26–34)	40.5 (33–48)	<0.001
Male/Female	6/4	4/6	7/3	0.387
Number of postgraduate months	0 (0–12)	36 (24–36)	126 (78–186)	< 0.001
Spinals performed in the last month	0 (0–1)	6.5 (1.7–20)	10 (5–10.5)	0.001
Spinals performed in the last 6 months	0 (0–1)	32.5 (27.5–42.5)	54 (27–60)	0.004

Values are expressed in median and interquartile range (Q1–Q3)

**Fig. 2:**
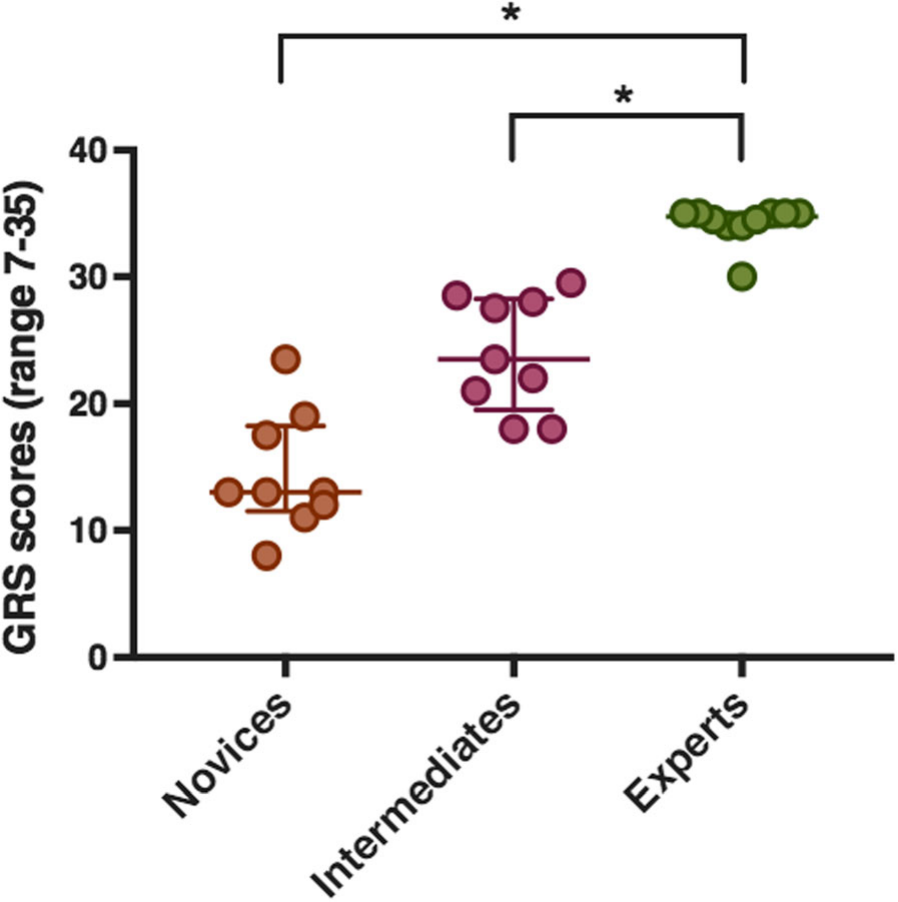
Global Rating Scale scores achieved by novices, intermediates and experts. Values are expressed in median and range (Q1–Q3). * Statistically significant at *P* < 0.05

The inter-rater reliability of the GRS scores between both evaluators had a Kappa coefficient of 0.76 (CI 0.58–0.92). Novice residents had significantly lower performance levels than experts during the procedure, expressed as lower scores in the total Global Rating Scale (Fig. 2). All groups had significant differences between them (Table 2). However, there was no difference in checklist scores between novices and intermediates and between intermediates and experts (Table 2).

**Table 2:** Visual assessment scales

	^A^ Novices (*n* = 10)	^B^ Intermediates (*n* = 10)	^C^ Experts (*n* = 10)	^AB^ *P* value	^BC^ *P* value	^AC^ *P* value	^ABC^ *P* value
Checklist scores (0–16)	12 (9–13)	12.5 (12–14)	12 (12–13)	NP	NP	NP	0.095
GRS scores (7–35)	13 (12–17.5)	23.5 (21–28)	34.75 (34–35)	0.15	0.02	< 0.001	< 0.001

*GRS* Global Rating Scale

*NP* Not performed (multiple comparisons were not performed when the overall test does not show significant differences across samples)

Values are expressed in median and interquartile range (Q1–Q3)

^ABC^ *p* value: *P* values obtained when comparing 3 groups with Kruskal Wallis test

^AB^ *p* value, ^BC^ *p* value, and ^AC^ *p* value: *P* values obtained when comparing columns with Dunn’s *post-hoc* test

The two phases of the procedure were analyzed separately. During preparation phase, total path length (TPL) travelled by both hands had statistically significant differences between novices and intermediates (*p* = 0.016) and between novices and experts (*p* = 0.018) (Table 2). The number of movements had significant difference only between novices and experts (*p* = 0.026). Likewise, the required time for preparation had significant differences between novices and intermediates (*p* = 0.02) and between novices and experts (*p* = 0.002). Nevertheless, motor skills measured with ICSAD (TPL, number of movements and time required) had no differences between intermediate and experts (Table 3).

**Table 3:** Preparation phase

	^A^ Novices (*n* = 10)	^B^ Intermediates (*n* = 10)	^C^ Experts (*n* = 10)	^AB^ *P* value	^BC^ *P* value	^AC^ *P* value	^ABC^ *P* value
Total path length (m)	40.17 (38.44–56.43)	32.9 (31.28–36.47)	30.32 (25.49–40.42)	0.016	1.0	0.018	0.006
Number of movements	256.5 (234.25–372.5)	223.5 (199.25–251.25)	186 (151.5–263.5)	0.181	1.0	0.026	0.025
Procedural time (s)	147.5 (131.75–183.25)	94.5 (86.25–115.75)	91 (82–117)	0.002	1.0	0.002	<0.001

Values are expressed in median and interquartile range (Q1–Q3)

^ABC^ *p* value: *P* values obtained when comparing 3 groups with Kruskal Wallis test

^AB^ *p* value, ^BC^ *p* value, and ^AC^ *p* value: *P* values obtained when comparing columns with Dunn’s *post-hoc* test for multiple comparisons

**Fig. 3:**
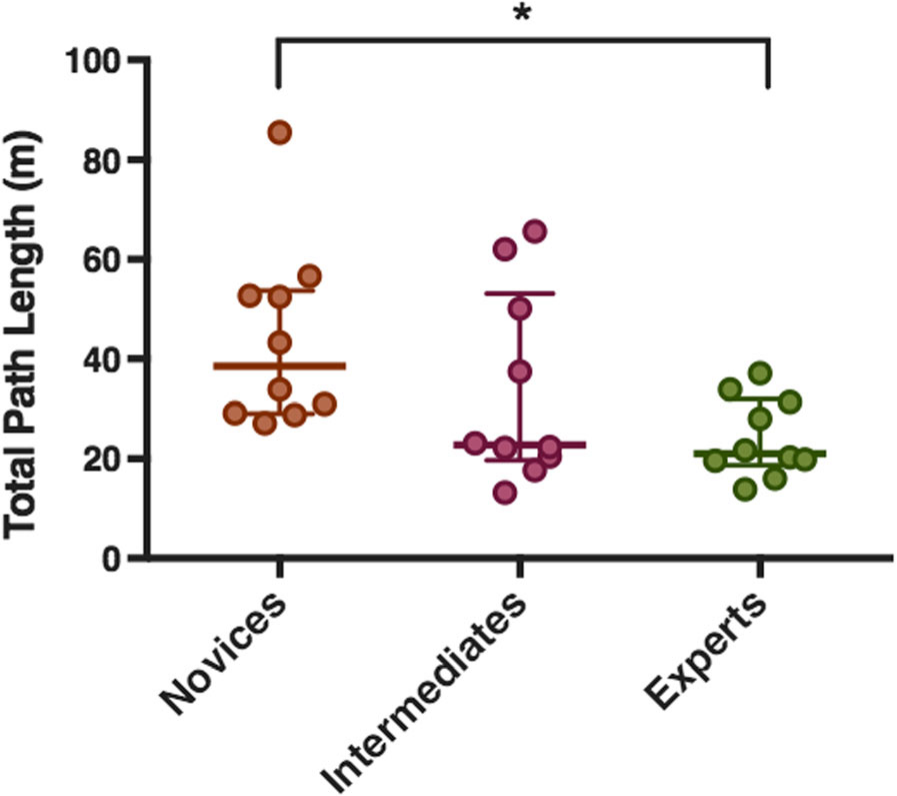
Distance traveled by both hands in meters by novices, intermediates and experts (Total Path Length). Values are expressed in median and range (Q1–Q3). Statistically significant at *p* < 0.05

During needling phase, the ICSAD data demonstrated that novice residents took a significantly longer time than expert staff anesthesiologists to complete the procedure [16]. Regarding the distance travelled by both hands during the procedure, novice residents had a significantly longer total path length than experts (*p* = 0.02). There was no difference between intermediate and experts (Fig. 3). Finally, the number of attempts done was different between groups (Table 4).

**Table 4:** Needling phase

	^A^ Novices (*n* = 10)	^B^ Intermediates (*n* = 10)	^C^ Experts (*n* = 10)	^AB^ *P* value	^BC^ *P* value	^AC^ *P* value	^ABC^ *P* value
Total path length (m)	38.5 (29.08–53.69)	22.75 (19.7–53.08)	21.03 (18.7–31.99)	0.34	0.79	0.02	0.026
Number of movements	193 (136.7–243)	125.5 (95.7–236)	114.5 (93–159.25)	0.18	1.0	0.05	0.045
Procedural time (s)	385.7 (195.8–639.57)	167.5 (107.32–414.72)	128.6 (98.7–159.65)	0.11	0.74	0.04	0.005
Number of attempts	14.5 (3–28)	5 (1–19)	1 (1–1)	0.187	0.714	0.007	0.009

Values are expressed in median and interquartile range (Q1–Q3)

^ABC^ *p* value: *P* values obtained when comparing 3 groups with Kruskal Wallis test

^AB^ *p* value, ^BC^ *p* value, and ^AC^ *p* value: *P* values obtained when comparing columns with Dunn’s *post-hoc* test

Spearman correlation coefficients between the three ICSAD dexterity measurements, against checklist and the global rating score are shown in Table 5. Spearman correlation coefficient between the total path length measured with ICSAD and the validated global rating scale scores demonstrated a moderate negative linear correlation, with a Spearman correlation coefficient of −0,46 (*p* = 0.012). This value means that the strength of association between both assessment tools is moderate. The negative sign in the correlation coefficient reflects an inverse relationship.

**Table 5:** Spearman correlation coefficients between the three ICSAD dexterity measurements, with checklist and global rating scale scores

	Checklist	*p* value	Global rating scale	*p* value
Total path length	- 0.13	0.5	- 0.46	0.012
Number of movements	- 0.19	0.32	- 0.41	0.028
Time	- 0.18	0.34	- 0.44	0.019

## Discussion

The results of this study demonstrate that ICSAD is a valid assessment tool of procedural skills involved in simulated spinal anesthesia. The three parameters delivered by ICSAD (TPL, number of movements and time required to complete the procedure) were significantly different when comparing novices and experts, confirming that this tool discriminates well between operators with those different levels of proficiency. Additionally, it correlates moderately well with the previous validated GRS.

There are several tools developed to assess technical skills, one of them are checklists. They are constructed upon a set of key actions defining a properly performed procedure [17]. In this study, we were unable to find differences between checklist scores obtained by novices and experts. Although it could be due to a type II error, a post Hoc analysis performed demonstrated an adequate level of power. Another possible explanation is that knowledge acquisition through the video reviewed prior to the assessment session allowed novices to complete easily the majority of the key actions requested by the checklist from a cognitive standpoint. This interpretation challenges the utility of checklists as an assessment tool for procedural competences. A systematic review of validity evidence for checklists versus global rating scales in simulation-based assessment supports this idea [18]. Although checklists have the appeal of a more objective measurement tool, evidence suggests that they do not necessarily confer greater validity and reliability [19].

Other options to assess motor skills are global rating scales (GRS). These instruments provide more information than a mere dichotomous type of output from checklists, being more suitable to grade operators’ performance. They have been shown to detect different levels of expertise more sensitively than the checklist [18]. For instance, in central venous catheter placement, Ma and colleagues provide an example whereby the use of a global rating scale (GRS) may be preferred over the use of two currently available checklists [19]. One of the main issues of GRS, however, is the fact that they rely upon subjective appreciations from observers. Additionally, these tools involve a higher cost because it requires a trained operator applying them.

Although these existing published tools currently assess many key elements, some domains continue to be under-represented. For pure motor skills, movement economy is considered a key difference between experts and novices [20]. In this context, besides time required to perform the task, other objective performance metrics, such as hand trajectory, velocity, acceleration, may provide additional performance assessment, with the aim to improve skill acquisition and transfer [6]. At the present time, there has been a change in the paradigm of how procedural skills are taught to trainees. There is now recognition that the traditional experience-based mode of acquiring technical skills should be replaced by a more structured competence-based method. In this context, more objective and quantitative measures could be a contribution to the training process. The capability of discriminating the degree of expertise of these types of tools may give some feedback and finally guide training, in order to detect the sufficient degree of competences acquired. To date, ICSAD has not been used yet to establish thresholds to be achieved with simulation based training curriculums for spinal anesthesia, neither to document acquisition of technical skills of national evaluation standards for bedside procedures.

Unexpectedly, ICSAD dexterities were not statistically different between novices and intermediates, and between intermediates and experts. Our “intermediates” and “experts” participants do not perform different in this procedure, having similar TPL and number of movements values. Hayter had similar findings when assessed epidural catheter insertion, proposing that they were not sufficiently powered to detect differences. Another possible theoretical explanation of the lack of differences between intermediates and experts is that most third year residents have already flattened their learning curves achieving enough proficiency to perform an “average” spinal anesthesia case unsupervised. This group of operators was categorized as intermediates because they were considered “experienced non-experts”, capable to perform well on routine problems applying the standard technique [21]. Following the five stage Dreyfus’ model of skill acquisition, we intended to have a “competent” group [22]. Hayter had similar findings when assessed epidural catheter insertion, proposing to add variables such as different patient ergonomic conditions, in order to discriminate in a more subtle way the level of expertise of advanced operators [7]. Since simulators try to resemble clinical scenarios with a standard level of difficulty (the average type of patient) it is possible that more difficult cases would be required to differentiate performances of intermediate and expert operators. This effect has been observed clearly in surgical scenarios [6]. Regarding the lack of difference between novices and intermediates, both groups performed different, but not significantly different. A possible interpretation is the failure of ICSAD as an assessment tool to differentiate between similar skill levels in a procedure such as spinal anesthesia. Spinal anesthesia is a shorter procedure with fewer steps in comparison with epidural and central venous catheter insertions, giving fewer chances to find any differences in performance. Another possible explanation for this situation is to be underpowered; however the post hoc analysis determined enough power to detect differences between groups. Finally, it is important to clarify that there are no previous data available to calculate a sample size on an “a priori” basis for this kind of studies, although other authors have been done with 20–30 participants [7, 8].

Our study has several limitations that require discussion. First, as we mentioned previously, it did not detect significant differences between novices and intermediates and between intermediates and experts. Second, our study was developed in a simulated environment. ICSAD has been previously validated as an assessment tool with similar protocols for lumbar epidural blocks [7] and ultrasound-guided supraclavicular blocks [8]. Both studies had established construct and concurrent validity of ICSAD in real patients; We decided to use a simulated torso for two main reasons: First, there are constraints in clinical practice related to ethical and safety issues; in this context, any deviation of training protocols are banned and the amount of error allowed to novice trainees must be minimal; even more, traditional approach for training residents implies that, if necessary, a permanent supervisor provide feedback or even intervene during the procedure affecting crude measurements and its subsequently validity and interpretation. Second, using simulators can standardize the level of difficulty for every operator, making the comparisons more reliable, especially among novices. It is precisely this rationalization that justifies the development of training programs in simulation for novices before their passage to clinical practice.

## Conclusions

As a conclusion, this study could validate ICSAD for assessing technical performance for spinal anesthesia in this simulated model. It could discriminate between expert and novices and has been correlated with previous validated global rating scale. Having as many as possible instruments for evaluating procedural skills could objectify better the process of acquisition of motor skills in novices. The contribution of this report is to set up objective data that could be essential to understand and design simulation-based training programs with reliable and objective goals to achieve and to provide statistical evidence that may be useful to improve simulation anesthesiology resident’s training protocols.

## Additional files

Additional file 1: Global Rating Scale for epidural anesthesia. (DOCX 133 kb)

Additional file 2: Procedural checklist for subarachnoid block. (DOCX 96 kb)

## Abbreviations

GRS: Global rating scale
ICSAD: Imperial College Surgical Assessment Device
TPL: Total path length
