# Supplementary material for: Validation of the imperial college surgical assessment device for spinal anesthesia
Source: BMC Anesthesiol. 2017 Sep 29;17:131. doi: 10.1186/s12871-017-0422-3 (PMC5622479; doi:10.1186/s12871-017-0422-3)
Supplement: Supplementary file 1 — Global Rating Scale for epidural anesthesia. (DOCX 133 kb) [file 12871_2017_422_MOESM1_ESM.docx]

Global Rating Scale for epidural anesthesia:

|  |  | **1** | **2** | **3** | **4** | **5** |
| --- | --- | --- | --- | --- | --- | --- |
| 1 | Preparation for procedure | Did not organize equipment well. Has to stop procedure frequently to prepare equipment |  | Equipment generally organized. Occasionally has to stop and prepare items |  | All equipment neatly organized prepared and ready for use |
| 2 | Respect for tissue | Frequently used unnecessary force on tissue or caused damage |  | Careful handling of tissue but occasionally caused inadvertent damage |  | Consistently handled tissues appropriately with minimal damage |
| 3 | Time and Motion | Many unnecessary moves |  | Efficient time/motion but some unnecessary moves |  | Clear economy of movement and maximum efficiency |
| 4 | Instrument Handling | Repeatedly makes tentative or awkward moves with instruments |  | Competent use of instruments but occasionally appeared stiff or awkward |  | Fluid moves with instruments and no awkwardness |
| 5 | Flow of procedure | Frequently stopped procedure and seemed unsure of next move seemed unsure of next move |  | Demonstrated some forward planning with reasonable progression of procedure |  | Obviously planned course of procedure with effortless flow from one move to the next |
| 6 | Knowledge of procedure | Deficient knowledge |  | Knew all important steps of procedure |  | Demonstrated familiarity with all aspects of procedure |
| 7 | Overall performance | Very poor |  | Competent |  | Clearly superior |
