# Supplementary material for: Validation of the imperial college surgical assessment device for spinal anesthesia
Source: BMC Anesthesiol. 2017 Sep 29;17:131. doi: 10.1186/s12871-017-0422-3 (PMC5622479; doi:10.1186/s12871-017-0422-3)
Supplement: Supplementary file 2 — Procedural checklist for subarachnoid block. (DOCX 96 kb) [file 12871_2017_422_MOESM2_ESM.docx]

Procedural checklist for subarachnoid block.

Task:

1. Performs a “time-out” and places monitors on patient (pulse oximetry and NIBP).
2. Verifies that spinal kit tray, nonsterile and sterile gloves (correct size), and cleansing solution.
3. Palpates the superior aspects of the iliac crests and identifies the intersection at the L4 spinous process with nonsterile gloves on. Marks position at the L3/L4 or L4/L5 interspace.
4. Cleans the overlying skin with chlorhexidine.
5. Opens the spinal tray before placing sterile gloves on.
6. Puts on sterile gloves with proper technique
7. Applies sterile drapes
8. Draws up lidocaine in the 3cc syringe and bupivacaine in the 5cc syringe. Administers local anesthesia in a wheal at the previously marked site.
9. Injects more anesthetic in the correct location and angle
10. Inserts the introducer needle in the middle of the interspace with a slight cephalad angulation of 10 to 15 degrees. The bevel of the spinal needle should be in the sagittal plane.
11. Advances spinal needle through anatomic structures until the subarachnoid space is reached. May experience a popping sensation as the ligament flavum is crossed.
12. Withdraws the stylet each time a pop is felt to assess for CSF flow.
13. Confirms CSF flow by aspiration before and after injecting anesthetic.
14. Removes the spinal and introducer needle together once completed.
15. Applies pressure with the provided 2 × 2 gauze and assesses good hemostasis.
16. Removes the drape, lays the patient, and observes vitals. Disposes of all sharps and biohazard material appropriately.
